# Genome-wide identification and expression analysis of SBP-like transcription factor genes in Moso Bamboo (*Phyllostachys edulis*)

**DOI:** 10.1186/s12864-017-3882-4

**Published:** 2017-06-27

**Authors:** Feng Pan, Yue Wang, Huanglong Liu, Min Wu, Wenyuan Chu, Danmei Chen, Yan Xiang

**Affiliations:** 1 Laboratory of Modern Biotechnology, School of Forestry and Landscape Architecture, Anhui Agricultural University, Hefei, 230036 China; 2 Key Laboratory of Crop Biology of Anhui Province, School of Life Sciences, Anhui Agricultural University, Hefei, 230036 China

**Keywords:** Moso bamboo, SPL genes, Transcription factor, Expression patterns

## Abstract

**Background:**

The *SQUAMOSA promoter binding protein*-*like* (SPL) proteins are plant-specific transcription factors (TFs) that function in a variety of developmental processes including growth, flower development, and signal transduction. SPL proteins are encoded by a gene family, and these genes have been characterized in two model grass species, *Zea mays* and *Oryza sativa*. The SPL gene family has not been well studied in moso bamboo (*Phyllostachys edulis*), a woody grass species.

**Results:**

We identified 32 putative *PeSPL* genes in the *P. edulis* genome. Phylogenetic analysis arranged the *PeSPL* protein sequences in eight groups. Similarly, phylogenetic analysis of the SBP-like and SBP proteins from rice and maize clustered them into eight groups analogous to those from *P. edulis*. Furthermore, the deduced *PeSPL* proteins in each group contained very similar conserved sequence motifs. Our analyses indicate that the *PeSPL* genes experienced a large-scale duplication event ~15 million years ago (MYA), and that divergence between the *PeSPL* and *OsSPL* genes occurred 34 MYA. The stress-response expression profiles and tissue-specificity of the putative *PeSPL* gene promoter regions showed that SPL genes in moso bamboo have potential biological functions in stress resistance as well as in growth and development. We therefore examined *PeSPL* gene expression in response to different plant hormone and drought (polyethylene glycol-6000; PEG) treatments to mimic biotic and abiotic stresses. Expression of three (*PeSPL10*, −*12*, −*17*), six (*PeSPL1*, −*10*, −*12*, −*17*, −*20*, −*31*), and nine (*PeSPL5*, −*8*, −*9*, −*14*, −*15*, −*19*, −*20*, −*31*, −*32*) genes remained relatively stable after treating with salicylic acid (SA), gibberellic acid (GA), and PEG, respectively, while the expression patterns of other genes changed. In addition, analysis of tissue-specific expression of the moso bamboo SPL genes during development showed differences in their spatiotemporal expression patterns, and many were expressed at high levels in flowers and leaves.

**Conclusions:**

The *PeSPL* genes play important roles in plant growth and development, including responses to stresses, and most of the genes are expressed in different tissues. Our study provides a comprehensive understanding of the *PeSPL* gene family and may enable future studies on the function and evolution of SPL genes in moso bamboo.

**Electronic supplementary material:**

The online version of this article (doi:10.1186/s12864-017-3882-4) contains supplementary material, which is available to authorized users.

## Background

Moso bamboo (*Phyllostachys edulis*), a large woody bamboo species that is widely distributed in Asia, possesses great economic, ecological, and cultural values. *P. edulis* accounts for almost 70% of the total area dedicated to bamboo cultivation in China, and is used in the manufacture of paper, timber, art wares, and also as a food resource [1]. As a perennial plant, moso bamboo is usually characterized by rapid growth and a long vegetative period before flowering [2]. Because SPL genes are known to regulate inflorescence branching and kernel development along with other major physiological processes, it is important to probe the underlying function of moso bamboo SPL (*PeSPL*) genes to understand the regulation of flowering in bamboo.

In addition, transcriptional control depends on TFs, which can regulate gene expression in response to stresses such as drought, cold, and salinity, as well as pathogen stimuli and phytohormones. Thus, TFs are involved in regulating defense responses and gene regulation networks in plant growth and development. The SPL genes encode a special family of TFs that are unique to plants [3]. SPL proteins contain a highly conserved DNA-binding domain, the SBP domain, which consists of approximately 79 amino acid residues that include ten conserved cysteine and histidine residues. The SBP domain comprises two zinc-binding sites and individual DNA-binding and nuclear localization domains [4, 5]. The two SPL genes (*AmSBP1* and *AmSBP2*) first isolated from the snapdragon, *Antirrhinum majus*, were identified based on their direct interaction with a promoter sequence motif in *SQUAMOSA*, a floral meristem identity gene [4]. The first SPL gene identified in *Arabidopsis* was *SPL3*, which was shown to promote flowering under long day conditions. In addition, *AtSPL3* binds to a conserved *cis*-element in the promoter region of *APETALA1*, a floral meristem identity gene that is an ortholog of *SQUA*, similar to the snapdragon genes *AmSBP1* and *AmSBP2* [6].

Previous biochemical and physiological studies have demonstrated that the SPL family of TFs have positive functions in controlling plant development. In *Arabidopsis*, all 16 SPL genes in the genome have been identified [7], and they play essential roles in leaf development [8], flower development [9], shoot development [10], sporogenesis [11], fertility [12], reproductive stage and nutritional changes [13], plant hormone signal transduction [14], and leaf primordia interval formation [15]. The SPL genes are also involved in the regulation of copper homeostasis [16] and the gibberellic acid (GA) response [17]. At present, the SPL gene family has been isolated, identified, and characterized in a number of plant species including silver birch [18], green alga [19], rice [20], moss [21], maize [22], *Populus trichocarpa* [23], tomato [24], grape [25], apple [26], melons [27]*, Salvia miltiorrhiza* [28]*,* castor bean [29], *Gossypium hirsutum* [30], *Prunus mume* [31], petunia [32], citrus [33], peanut [34], chrysanthemum [35], and pepper [36]. However, the SPL proteins in moso bamboo have not been studied, and their functions are unclear at present.

With the rapid development of high-throughput DNA sequencing technologies, genome sequencing has provided us with an opportunity to perform a genome-wide analysis of the SPL genes in moso bamboo. In this study, we conducted the first systematic, comprehensive analysis of the SPL genes in *Phyllostachys edulis*. We identified 32 putative *PeSPL* transcription factor genes, and systematically analyzed their structure, phylogenetic relationships, predicted conserved motifs, evolutionary patterns and divergence, *cis*-elements, and expression levels in response to different treatments and in five different organs.

## Methods

### Database searches for moso bamboo SPL genes

To identify the SPL gene members in moso bamboo, the Hidden Markov Model (HMM) profiles of all sequences containing an SBP domain (PF03110) were used to search the National Center for Gene Research database (http://www.ncgr.ac.cn/bamboo) [37]. In addition, in order to verify the identity of these putative *PeSPLs*, the non-redundant candidate SPL genes in moso bamboo were identified using the InterproScan program to confirm the existence of the conserved SBP domain, and all of the SBP-like genes without an SBP domain were discarded [38]. Information for the *PeSPL* genes and predicted proteins, including CDS lengths, the predicted number of amino acids, and physicochemical parameters were obtained from the Bamboo GDB (http://www.bamboogdb.org).

### Phylogenetic analyses and intron-exon structure determination

To examine the domain organization of the predicted SBP proteins in moso bamboo in detail, multiple sequence alignments of SBP domain-containing sequences were performed using Clustal W software [39], and we constructed a phylogenetic tree based on the complete *PeSPL* sequences using the N-J method as implemented in MEGA software (v5.1) [40] with a bootstrap analysis of 1000 replicates. The combined phylogenetic tree of *OsSPL, ZmSPL* and *PeSPL* protein sequences was generated using the same method. In addition, the predicted exon-intron structures of the *PeSPL* genes were visualized using the online Gene Structures Display Server (http://gsds.cbi.pku.edu.cn) by comparing the cDNA with the corresponding genomic DNA sequences.

### Identification of conserved protein sequence motifs

Conserved motifs present in the *PeSPL* proteins were identified with the online MEME tool (http://meme.sdsc.edu/meme/intro.html) using the default parameter settings: maximum number of motifs = 20; optimum motif length range between 6 and 200 [41]. We also used the Pfam (http://pfam.sanger.ac.uk/search) [42] and SMART (http://smart.embl-heidelberg.de/) databases to annotate the structural motifs [43].

### Identification of paralogs and rice orthologs in moso bamboo

We used BLASTn [44, 45] to perform all-against-all nucleotide sequence similarity searches of the transcribed SPL sequences to identify paralogous sequences as shown by Blanc and Wolfe [44]. Sequences that showed at least 40% identity with aligned regions >300 bp were defined as paralogs. Putative rice orthologs were identified by using each sequence as a query to search against all sequences from moso bamboo. If the SPL gene sequences gave the best hit, and >300 bp of the two sequences aligned, the two genes were then defined as being orthologous [46].

### Calculation of Ka/Ks values

Pairwise alignments of the paralogous and orthologous SPL gene sequence pairs were performed with ClustalX 2.11, and the results were further analyzed using MEGA 6.0. A synonymous substitution (Ks) is defined as a mutation in which a nucleotide base is replaced by a different base in a protein-coding region of a gene that does not result in an amino acid change in the encoded protein, while a non-synonymous substitution (Ka) results in a change in the amino acid sequence of a protein [47]. The non-synonymous and synonymous substitution rates were then calculated using DnaSP 5 to analyze gene duplication events [48, 49]. As described by Lin et al. (2014) Ks can be used as a proxy for time when dating large-scale duplication events [50]. Therefore, the date of duplication events was subsequently converted into divergence time (T) using the formula T = Ks/2λ × 10^−6^ Mya for each gene pair. Based on previous studies, the approximate value of the clock-like synonymous substitution rate (λ) was 6.5 × 10^−9^ years for both moso bamboo and rice [37, 51].

### Analysis of the putative promoter regions of the *PeSPL* genes

To identify the *cis*-elements in the putative promoter regions, we examined the 2000 bp upstream sequences of the *PeSPL* genes. We used the PLACE website (http://www.dna.affrc.go.jp/PLACE/signalscan.html) [52] and Plant-CARE (http://bioinformatics.psb.ugent.be/webtools/plantcare/html/) [53] to identify the predicted *cis*-regulatory elements present in the gene promoters.

### Plant growth conditions and seedling treatments

The treatment-induced gene expression profiles of 32 *PeSPL* genes were examined in young leaves of three-month old seedlings of moso bamboo grown from seeds collected from Guilin in Guang Xi Province, China. All seeds were provided and identified by the Guilin Forestry Bureau. The moso bamboo seeds were germinated in culture dishes on moist, sterile filter paper at 25 °C in the dark. The seedlings were then moved into plastic pots containing a mixture of black soil and vermiculite and grown in a greenhouse under 14 h of light (from 07:30 to 21:30) at 24–28 °C and 80% humidity. These seedlings were then used in experiments to assay gene expression in response to three stress conditions. The moso bamboo seedlings were watered with Hoagland’s nutrient solution twice a week. The treatments were performed by spraying the young moso bamboo leaves individually with 100 μM gibberellin (GA), 20% polyethylene glycol-6000 (PEG) solution, and 100 μM salicylic acid (SA). The young leaves from the stress-treated plants were collected at 1, 3, 6, 12, and 24 h after treatment. Untreated seedlings were used as the control groups. Also, tissue-specific transcription profiles of 32 *PeSPL* genes were analyzed in various vegetative and reproductive tissues in plants collected from Ningguo, Anhui Province, China. In addition, the permission of tissues collection and identification for the experiments were obtained from Jianguo Pei of Ningguo Forestry Bureau. The samples included various tissues (young leaves, mature leaves, roots, shoots, and panicles). After gathering, the plant tissues were immediately frozen in liquid N_2_ and stored at −80 °C prior to RNA extraction.

### Microarray-based expression analysis

We performed a comprehensive expression profile of the *PeSPL* genes in order to reveal their function in moso bamboo growth and development. The data was obtained from the NCBI Short Read Archive (SRA) database. The unprocessed RNA-seq reads from BioProject ERP001341 were then pruned to eliminate low quality base-calls (Q < 20) and adaptor sequences using the pipeline Fastq clean [54]. The clean paired reads were mapped to the *Phyllostachys edulis* reference genome using the pipeline tophat2 with the default parameters. Briefly, TopHat2 uses Bowtie2 as an alignment ‘engine’ and breaks reads that Bowtie2 can not align on its own into segments [55]. And then, the differences in gene expression were tested with Cufflinks [55]. The heatmap of *PeSPL* gene expression was drawn with the Heatmapper Plus tool (http://www.bar.utoronto.ca/ntools/cgi-bin/ntools_heatmapper.cgi) for seven moso bamboo tissues and different developmental stages (leaf, early panicle, advanced panicle, root, rhizome, 20-cm shoot, and 50-cm shoot) [56].

### RNA isolation and qRT-PCR analysis

Total RNA was extracted from frozen young leaf tissue from different stress treatments and different organs of moso bamboo with TRIzol reagent (Invitrogen, Ca, USA) as directed by the manufacturer. We synthesized first-strand cDNA using the Prime-Script™ RT Reagent Kit (TaKaRa) according to the manufacturer’s instructions. We then designed 32 pairs of gene-specific primers using Primer Express 3.0. Primer specificity was checked by BLAST searches using data from a local CDS database downloaded from BambooGDB (http://www.bamboogdb.org/page/microrna.jsp). In this study, the *TIP41* (tonoplast intrinsic protein 41) gene was used as a reference for normalization because it has a stable expression pattern [57]. qRT-PCR amplifications were performed on an ABI 7300 Real-Time system (Applied Biosystems) in 20 μl reactions containing 1 μl of each gene-specific primer, 1 μl of cDNA sample, 7 μl ddH_2_O, and 10 μl SYBR Green Master Mix reagent (Applied Biosystems). All primers for amplification of *PeSPL* genes are given in Additional file 1: Table S1. The qRT-PCR amplification conditions were: 95 °C for 30 s, followed by 40 cycles of 95 °C for 10 s, 55 °C for 15 s, and 72 °C for 10 s. A melting curve analysis was performed for each sample to verify the specificity of the reactions. There were three biological and three technical replicates performed for each sample. The relative expression levels were evaluated using the ΔΔCT method. It is worth noting that for the stress treatments, relative gene expression [2^−ΔΔCT, CK (0 h)^] for each gene in the control plants was normalized to 1 as described previously [58].

## Results

### Identification and characterization of SPL family genes in moso bamboo

In this study, we identified 32 SPL genes in moso bamboo after removing redundant sequences from further analysis. We named the 32 SPL genes *PeSPL1* to *PeSPL32* based on their chromosomal locations from top to bottom. All of these sequences were located, and the details of the SPL gene family in moso bamboo is given in Table 1. The predicted *PeSPL* proteins varied greatly with respect to molecular weight and length. The moso bamboo SPL genes encode proteins ranging from 177 (*PeSPL18*) to 1071 (*PeSPL30*) amino acids (aa) in length, and from 18,838.3 (*PeSPL18*) to 118,663 (*PeSPL30*) kDa in molecular weight. The other characteristics of the individual SPL genes, including isoelectric point (pI) and the number of predicted exons, are also given in Table 1.

**Table 1:** Gene names, genomic information, and predicted protein parameters for the 32 putative *PeSPL* genes in the moso bamboo genome

Name	Gene ID	Chr	Location	CDS length(bp)	Protein			Exons
					Size (aa)	MW(kDa)	pI	
PeSPL1	PH01000002G1660	PH01000002	PH01000002:1,200,472–1,205,728(+ stand)	1278	425	46,157.1	8.45	4
PeSPL2	PH01000040G1230	PH01000040	PH01000040:816,810–825,960(+ stand)	2850	949	103,586	5.73	11
PeSPL3	PH01000050G0170	PH01000050	PH01000050:85,280–89,502(+ stand)	1080	359	38,147.5	8.99	4
PeSPL4	PH01000057G0060	PH01000057	PH01000057:47,613–53,685(+ stand)	1044	347	38,395.5	8.39	3
PeSPL5	PH01000095G1560	PH01000095	PH01000095:1,058,556–1,062,174(+ stand)	987	328	35,561.9	8.63	4
PeSPL6	PH01000117G1390	PH01000117	PH01000117:964,573–972,149(− stand)	1236	411	43,399.7	7.97	3
PeSPL7	PH01000150G0460	PH01000150	PH01000150:291,251–295,678(+ stand)	1071	356	36,355.1	7.24	3
PeSPL8	PH01000164G0630	PH01000164	PH01000164:351,140–355,432(+ stand)	1389	463	50,159.3	9.42	4
PeSPL9	PH01000176G0670	PH01000176	PH01000176:478,695–482,876(− stand)	1455	484	52,719.5	9.31	5
PeSPL10	PH01000450G0480	PH01000450	PH01000450:319,332–324,328(+ stand)	1572	523	56,864.4	8.84	7
PeSPL11	PH01000548G0610	PH01000548	PH01000548:433,324–435,597(− stand)	912	303	32,689.6	6.61	3
PeSPL12	PH01000608G0740	PH01000608	PH01000608:478,612–483,867(+ stand)	1287	428	45,044.4	7.68	4
PeSPL13	PH01000770G0270	PH01000770	PH01000770:236,104–237,441(+ stand)	939	312	34,173.7	9.09	1
PeSPL14	PH01000915G0320	PH01000915	PH01000915:189,707–197,542(− stand)	3186	1061	116,841	6.91	11
PeSPL15	PH01000920G0420	PH01000920	PH01000920:317,552–324,017(+ stand)	2076	691	76,451.9	5.85	11
PeSPL16	PH01000969G0180	PH01000969	PH01000969:168,903–175,012(+ stand)	879	292	32,015.7	6.35	3
PeSPL17	PH01000985G0610	PH01000985	PH01000985:379,393–386,488(− stand)	2865	954	104,628	5.53	11
PeSPL18	PH01001167G0360	PH01001167	PH01001167:221,659–223,521(− stand)	534	177	18,838.3	8.61	2
PeSPL19	PH01001316G0020	PH01001316	PH01001316:5985–15,805(− stand)	2427	808	88,965.7	5.34	11
PeSPL20	PH01001685G0440	PH01001685	PH01001685:285,382–288,941(− stand)	585	194	21,258.3	8.57	3
PeSPL21	PH01001751G0380	PH01001751	PH01001751:279,930–283,848(− stand)	1008	335	36,542.2	9.05	4
PeSPL22	PH01001834G0370	PH01001834	PH01001834:290,182–294,145(− stand)	1236	411	43,721.6	8.73	3
PeSPL23	PH01001853G0340	PH01001853	PH01001853:289,935–292,481(− stand)	969	322	34,303.1	8.56	3
PeSPL24	PH01001910G0100	PH01001910	PH01001910:76,552–80,372(+ stand)	1044	347	38,418.6	8.73	3
PeSPL25	PH01002673G0070	PH01002673	PH01002673:47,490–53,632(+ stand)	1203	400	42,694.8	7.57	3
PeSPL26	PH01002789G0180	PH01002789	PH01002789:101,788–107,846(− stand)	966	321	34,363.5	6.93	3
PeSPL27	PH01003044G0090	PH01003044	PH01003044:55,489–58,672(+ stand)	585	195	20,422.9	9.62	2
PeSPL28	PH01003178G0220	PH01003178	PH01003178:102,673–108,004(− stand)	1155	384	41,253.5	9.44	3
PeSPL29	PH01003773G0220	PH01003773	PH01003773:115,930–124,043(+ stand)	1284	427	45,833.1	8.59	4
PeSPL30	PH01004096G0180	PH01004096	PH01004096:90,610–97,402(− stand)	3216	1071	118,663	6.13	11
PeSPL31	PH01004816G0090	PH01004816	PH01004816:49,108–52,984(+ stand)	993	331	35,967.5	8.96	4
PeSPL32	PH01007654G0010	PH01007654	PH01007654:454–3458(+ stand)	963	320	35,200.1	8.92	5

### Phylogenetic analysis of the *PeSPL* genes

**Fig. 1:**
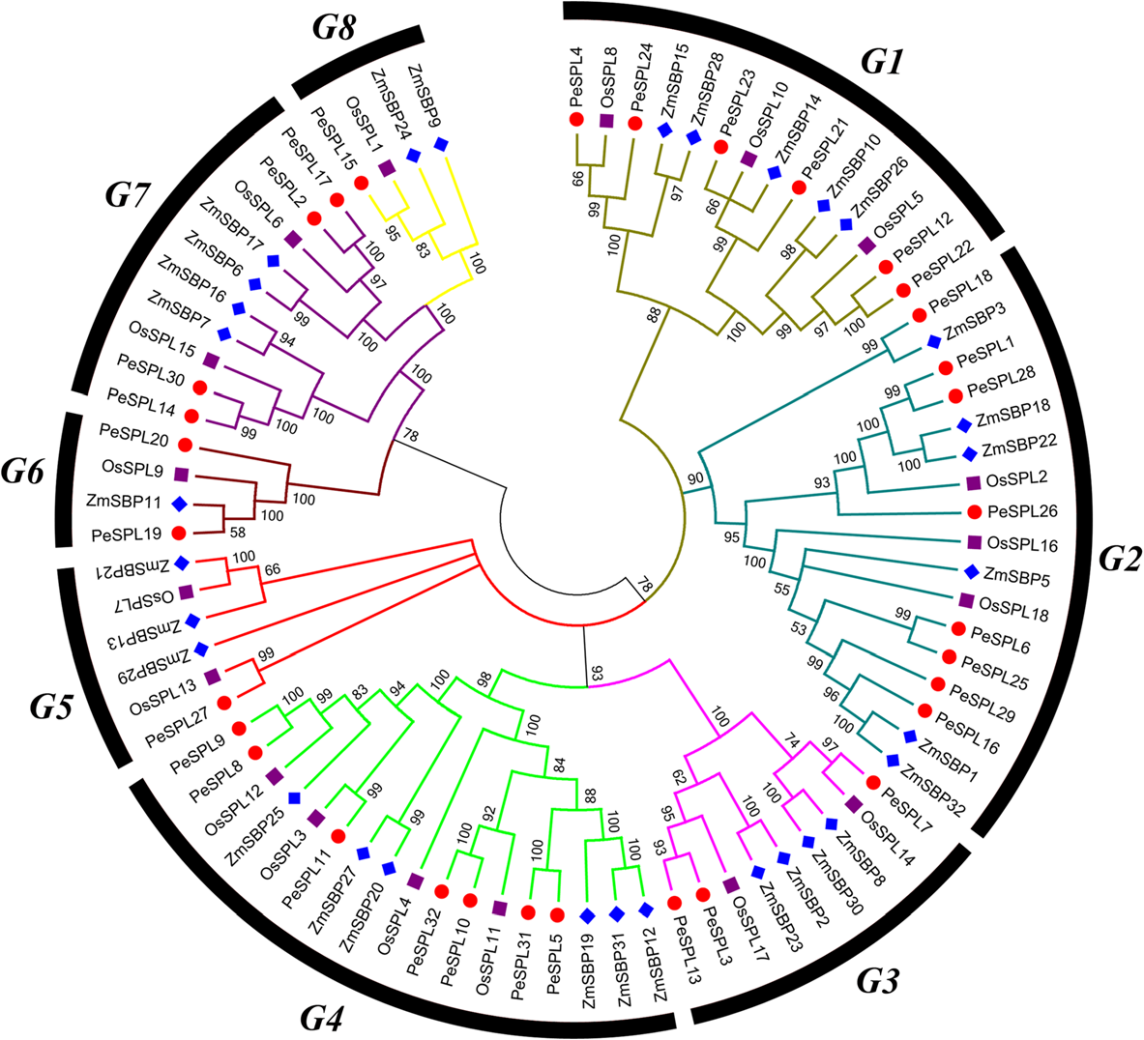
Phylogeny and distribution of SPL protein from three plant species. Phylogenetic tree of SPL proteins from rice, maize and moso bamboo. The tree was generated with Clustal X 2.0 software using the neighbour-joining method

The functions of some SPL genes have been characterized in rice (*Oryza sativa*), a model species in the botanical family *Poaceae*. To examine the inferred evolutionary relationships between the SBP domain-containing proteins in the grass family, we constructed a phylogenetic tree from alignments of the full-length SPL protein sequences for members of three major Poaceae subfamilies; the *Bambusoideae* (*Phyllostachys edulis*), *Ehrhartoideae* (rice), and *Panicoideae* (*Zea mays*) (Fig. 1). The *OsSPL* and *ZmSBP* sequences were obtained, and we generated a phylogenetic tree using the Neighbor-joining (NJ) algorithm in MEGA software (v5.1) with the *PeSPL* sequences. The phylogenetic tree contains 82 putative SPL protein sequences from the three monocot species; 18 from the *Oryza sativa*, 32 from *Zea mays*, and 32 from *P. edulis.* The detailed characteristics of the SPL genes from rice and maize are given in Additional file 1: Table S2. The phylogenetic tree showed that the predicted SPL proteins clustered into eight groups, G1- G8, as described previously (Fig. 1).

### Determination of exon-intron structure and identification of conserved sequence motifs in moso bamboo SPL genes

**Fig. 2:**
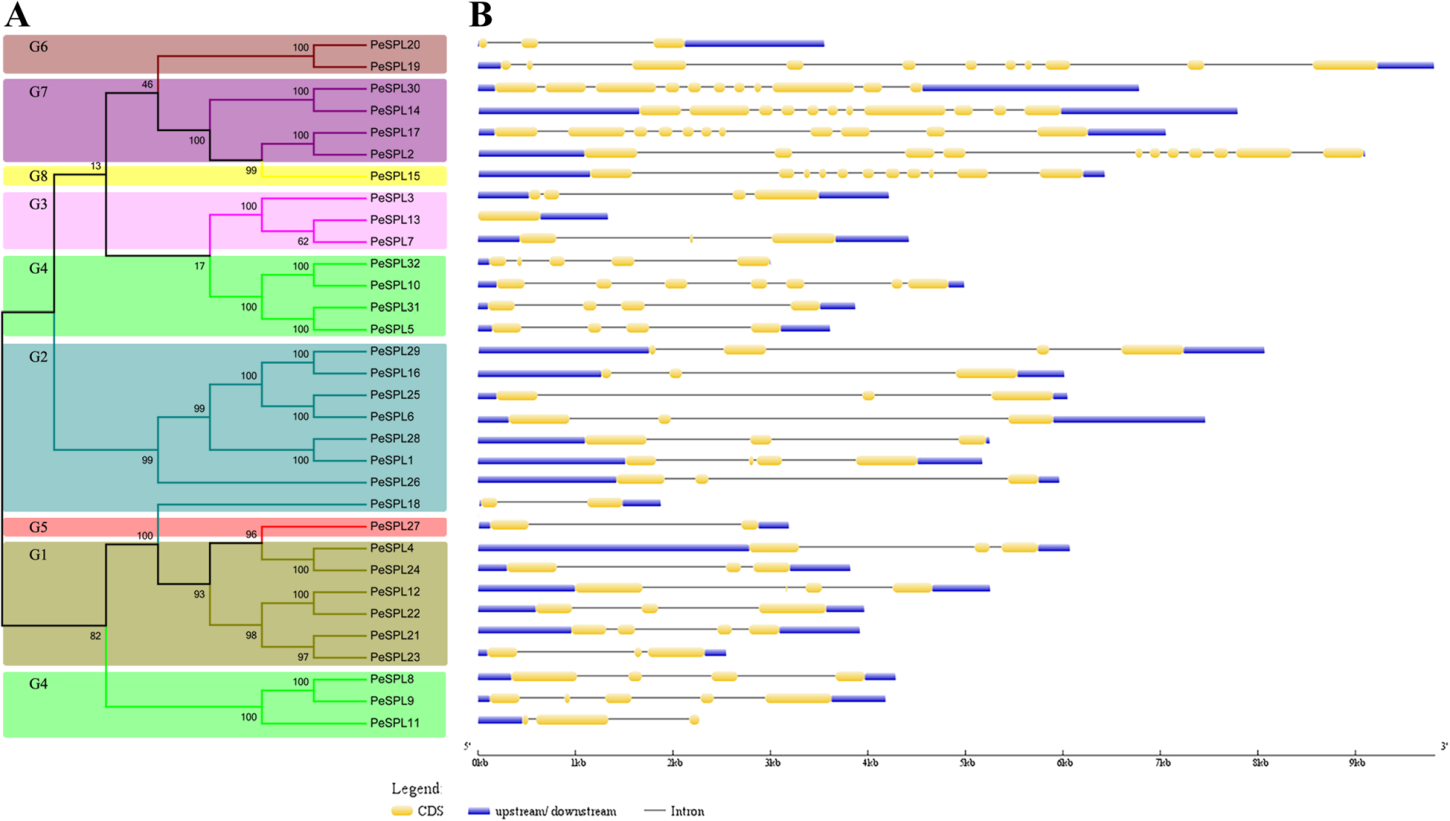
The unrooted tree of *PeSPL* genes family and exons-introns organization of moso bamboo (*Phyllostachys edulis*) SPL proteins. **a** The number of above or below branches of the phylogenetic tree indicate bootstrap values. Bootstrap values from 1000 replicates are indicated at each node. The unrooted tree was constructed with Clustal X2.0 by the Neighbor-Joining (NJ) method with 1000 bootstrap replicates. The *middle set* of numerals on the tree represented eight *PeSPL* groups. **b** *Yellow rectangles* represent exons, *grey lines* represent introns and *blue boxes* represent Untranslated regions (UTRs)

We examined the structural diversity of the SPL gene family in moso bamboo by constructing a phylogenetic tree based on the full-length predicted *PeSPL* protein sequences. This analysis also grouped these proteins into eight clusters, and agrees well with the description given above for the three plant species (Figs. 1 and 2a). A possible mechanism driving the evolution of multigene families involves genetic structural diversity. We compared the numbers, lengths, and arrangement of the exons and introns in the gene sequences (Fig. 2b) to gain further insight into the structural diversity of the *PeSPL* genes. As shown in Fig. 2, the genes in group 7, group 8, and *PeSPL19* in group 6 contain the largest number of exons (11). The other 26 genes contain between 1 and 7 exons. The results also show that *PeSPL13* in group 3 has no introns or upstream sequences. In addition, the *PeSPL11* gene in group 4 has no downstream sequence. An interesting feature of this analysis is that five sister gene pairs (*PeSPL30*/*−14*, *PeSPL17*/*−2*, *PeSPL31*/*−5*, *PeSPL25*/*−6* and *PeSPL4*/*−24*) were found to have the same intron phase and intron/exon number. However, the intron lengths showed considerable length variation in these five sister gene pairs. We also identified some differences; for example, eight sister gene pairs (*PeSPL20*/*−19*, *PeSPL13*/*−7*, *PeSPL32*/*−10*, *PeSPL29*/*−16*, *PeSPL28*/*−1*, *PeSPL12*/*−22*, *PeSPL21*/*−23* and *PeSPL8*/*−9*) varied greatly with respect to their structural organization and the numbers of introns and exons.

**Fig. 3:**
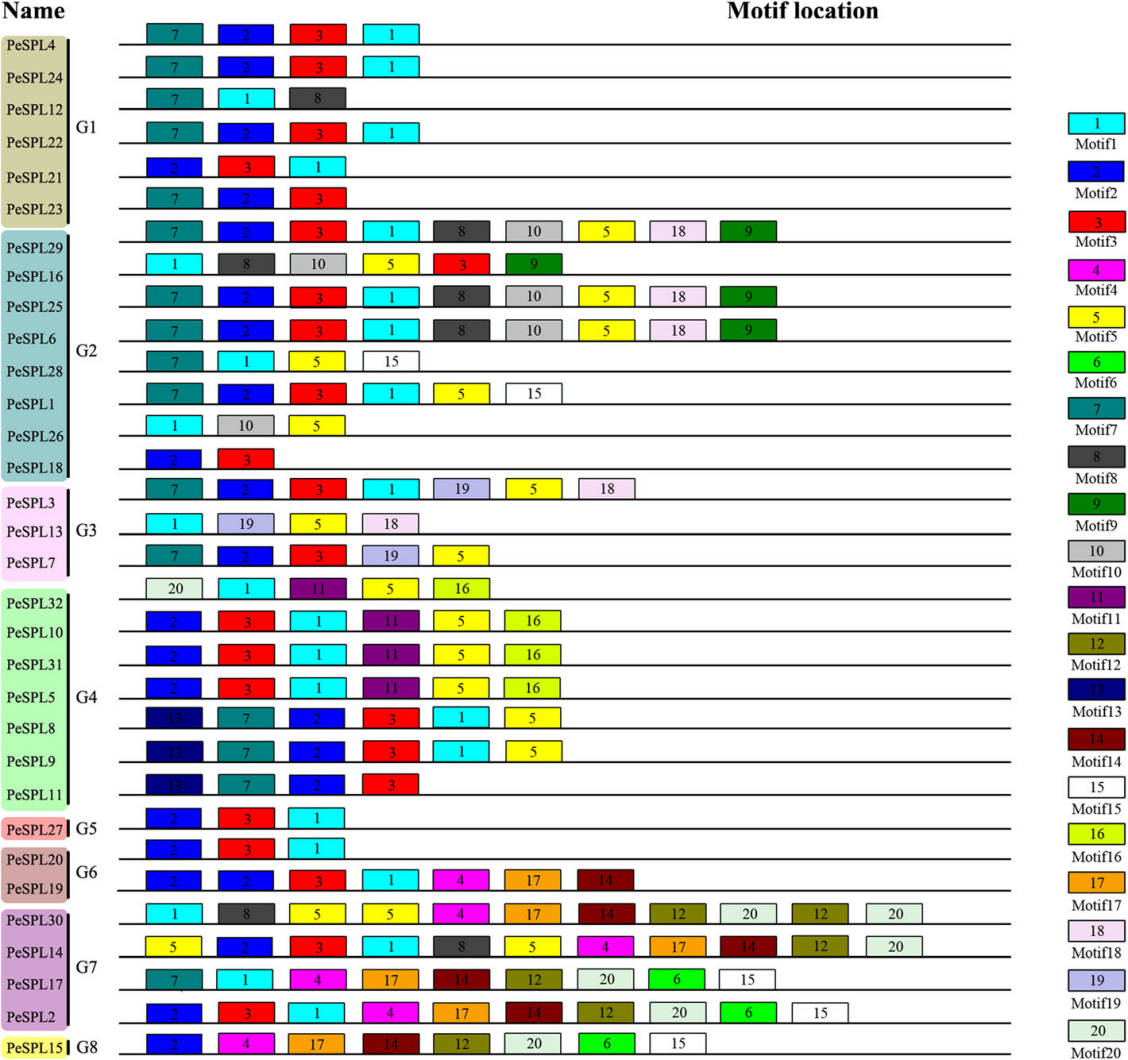
Schematic representation of the 20 conserved motifs in *PeSPL* proteins. Motifs of the *PeSPL* proteins were identified by MEME online tool. Each motif was represented by different *coloured block*, with their numbers in the centre of the motifs. The *number in boxes* (1–20) represents motif 1 – motif 20, respectively. The position and length of each *coloured box* represents the actual motif size

We initially searched for conserved sequence motifs using the MEME web server to further understand the compositions and diversity of motifs present in the predicted *PeSPL* proteins. A total of 20 distinct motifs were identified and designated motif 1 to motif 20 (Fig. 3); the details of the conserved amino acid sequences and their lengths are shown in Additional file 1: Table S3. It is clear that some of the gene pairs (*PeSPL4*/−*24*, *PeSPL25*/−*6*, *PeSPL31*/−*5*, *PeSPL8*/−*9*, *PeSPL6*/−2*9*, *PeSPL25*/−2*9*) share complete motif profiles (Fig. 3), and that the sister gene pairs in general contain very similar conserved structural motifs. In addition, it is very interesting that some motifs were found to be specific to only one or two groups of *PeSPL* proteins. For instance, motifs 9 and 10 are found exclusively in group G2, and motif 19 is only present in group G3.

### Patterns of evolution and divergence in the SPL gene family between moso bamboo and rice

**Fig. 4:**
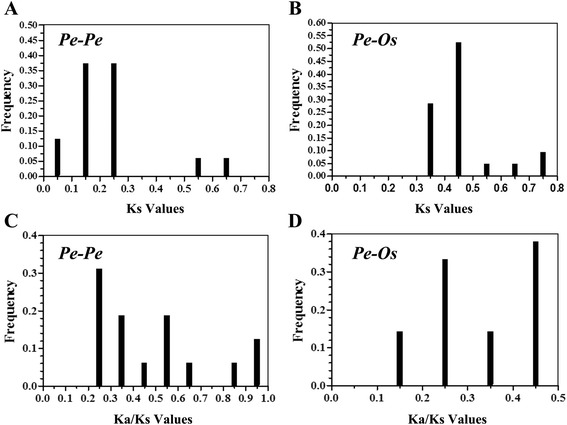
Ks and Ka/Ks value distribution of the SPL genes in the genomes of moso bamboo paralogous gene-pairs (*Pe*-*Pe*) and orthologous gene-pairs between moso bamboo and rice viewed through the frequency distribution of relative Ks and Ka/Ks modes. Distribution of Ks and Ka/Ks values were obtained from paralogous gene-pairs (*Pe*-*Pe*) in the moso bamboo genome (**a** and **c**), and orthologous gene-pairs between moso bamboo and rice (**b** and **d**)

Our analysis identified 16 putative paralogous gene pairs (*Pe*-*Pe*) in the moso bamboo genome and 21 ortholog pairs (*Pe*-*Os*) between the *PeSPL* and *OsSPL* genes using BLASTn sequence similarity analyses. All of the paralogous and orthologous pairs are listed in Table 2. For every SPL gene pair, we calculated the Ka/Ks ratios to estimate divergence times for the duplicated SPL genes (see Additional file 1: Tables S4 and S5). To further study divergence times between rice and moso bamboo, we also performed a statistical analysis of the Ka/Ks ratios and the Ks values. The frequency distributions of the Ka/Ks and Ks values for the orthologous and paralogous gene pairs from the rice and moso bamboo genomes were calculated (Fig. 4). The distribution of the calculated Ks values for the paralogous pairs in moso bamboo averaged ~0.2 (Fig. 4a), indicating that the SPL genes experienced a large-scale duplication event approximately 15 million years ago (MYA). A previous study estimated the timing of a whole-genome duplication in moso bamboo at 7–12 MYA [37], which could indicate that the large-scale duplication of the SPL genes occurred earlier. Also, for the rice-moso bamboo orthologous pairs, the major peak at 0.45 (Fig. 4b) shows that for the SPL genes the divergence time is 34 MYA. A comparison with the previous study that concluded that the divergence time between moso bamboo and rice was 7–15 MYA indicates that the SPL genes underwent gene evolution prior to the separation of the two progenitor species [59]. The Ka/Ks peaks in the moso bamboo genome are distributed between 0.2–0.3 (Fig. 4c), while the Ka/Ks ratios between the rice and moso bamboo genomes are distributed at 0.4–0.5 (Fig. 4d) [59]. This suggests that there was strong purifying selection in the SPL genes between the moso bamboo and rice genomes, as well as for the paralogs in the moso bamboo genome.

**Table 2:** Paralogous (*Pe-Pe*) and orthologous (*Pe-Os*) SPL gene pairs in moso bamboo and rice

*Pe-Pe*	*Pe-Os*
*PeSPL1/PeSPL28*	*PeSPL15/OsSPL1*
*PeSPL31/PeSPL5*	*PeSPL9/OsSPL3*
*PeSPL30/PeSPL14*	*PeSPL11/OsSPL3*
*PeSPL29/PeSPL6*	*PeSPL31/OsSPL4*
*PeSPL29/PeSPL25*	*PeSPL5/OsSPL4*
*PeSPL29/PeSPL16*	*PeSPL2/OsSPL6*
*PeSPL23/PeSPL21*	*PeSPL17/OsSPL6*
*PeSPL20/PeSPL19*	*PeSPL24/OsSPL8*
*PeSPL17/PeSPL2*	*PeSPL4/OsSPL8*
*PeSPL12/PeSPL22*	*PeSPL19/OsSPL9*
*PeSPL10/PeSPL32*	*PeSPL20/OsSPL9*
*PeSPL8/PeSPL9*	*PeSPL9/OsSPL12*
*PeSPL7/PeSPL13*	*PeSPL8/OsSPL12*
*PeSPL6/PeSPL25*	*PeSPL7/OsSPL14*
*PeSPL4/PeSPL24*	*PeSPL14/OsSPL15*
*PeSPL3/PeSPL13*	*PeSPL30/OsSPL15*
	*PeSPL25/OsSPL18*
	*PeSPL6/OsSPL18*
	*PeSPL29/OsSPL18*
	*PeSPL13/OsSPL17*
	*PeSPL3/OsSPL17*

### Analysis of putative promoter regions in the *PeSPL* gene family

Gene expression patterns and/or tissue specificity in response to stress are largely determined by *cis*-regulatory elements [60], and *cis*-regulatory elements in the promoter regions are closely correlated with multi-stimulus responsive genes [61, 62]. Four types of *cis*-elements containing a dehydration-responsive element, a TCA element involved in regulation of SA-related genes, a gibberellic acid response element (GARE) motif, and tissue-specific and development-related elements (Additional file 1: Table S6) were detected in current studies. This leds us to search the promoter regions of the moso bamboo SPL genes for possible stress-responsive and tissue-specific *cis*-elements using the PLACE and PlantCARE databases. *Cis*-elements can be located 2000-bp upstream of the promoter sequences and have practical impact on binding to target genes [63, 64]. Therefore, we searched the 2000-bp upstream sequences to identify putative *cis*-regulatory elements. We found many stress- and development-related elements in the promoter regions of the *PeSPL* genes. For example, there are ~20 drought-stress elements (S000415) in the *PeSPL24* promoter region, and the *PeSPL10* promoter contains as many as 12 tissue-specific elements. In addition, many genes have one or two SA and gibberellin responsive elements. Thus, further analysis of the putative promoter regions of *PeSPL* family genes helps to advance our understanding of stress tolerance and tissue-specificity in moso bamboo. Our results provide an indication that most SPL genes may function to enhance stress resistance, and are related to the developmental pathway.

### Differential expression profiling of SPL genes in moso bamboo tissues

In order to study the dynamics of *PeSPL* gene expression, we examined the gene expression profiles in different organs using high-throughput RNA sequencing (RNA-seq), which is a next-generation sequencing technology that provides a snapshot of gene expression profiles and mRNA levels at a given time [65, 66]. The RNA-seq reads can be aligned to the draft genome sequence of moso bamboo [37]. Previous studies mainly focused on the expression profiles in different tissues [67–69]. However, the expression profiles of *PeSPL* genes are not well characterized at present.

**Fig. 5:**
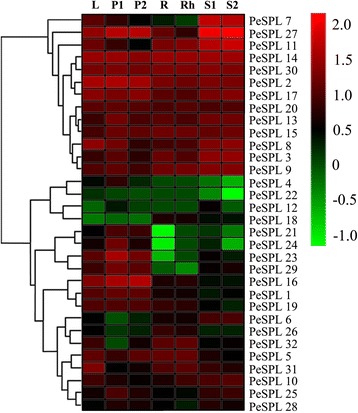
Expression profiles of *PeSPL* genes across different tissues and development stages. Heatmap showing hierarchical clustering of 32 SPL genes across different tissues analyzed. Color scale erected vertically at the *right side* of the picture represents log10 expression values, *green* indicates lower and *red* higher transcript abundance compared to the relevant control. Differential transcription of *PeSPL* genes. L: leaf; P1: early panicle; P2: advanced panicle; R: root; Rh: rhizome; S1: 20-cm shoot; S2: 50-cm shoot

We used microarray analysis to estimate the expression level of each *PeSPL* gene in different plant organs. The microarray data for 32 *PeSPL* genes (Additional file 1: Table S7) was downloaded from the NCBI database, and a heatmap was generated (Fig. 5).

The heatmap showed that six gene pairs (*PeSPL2/−17, PeSPL4/−24, PeSPL5/−31, PeSPL6/−25, PeSPL8/−9* and *PeSPL14/−30*) from the previously identified 16 paralogs in our study displayed distinct expression profiles in different developmental stages or different organs, revealing the different evolutionary fates of duplicated genes. For example, *PeSPL25* was expressed at high levels in the early panicle (P1) and advanced panicle (P2), while its counterpart *PeSPL6* had a relatively low expression level. However, the remaining 16 paralogous gene pairs had the same or similar relative expression levels. It is worth noting that 11 genes (*PeSPL2, PeSPL3, PeSPL8, PeSPL9, PeSPL13, PeSPL14, PeSPL15, PeSPL17, PeSPL20, PeSPL27*, and *PeSPL30*) showed high expression levels in these seven organs or developmental stages, suggesting that SPL genes play vital roles in plant development and growth.

### Differential expression profiling of *PeSPL* genes

Plants often experience a variety of environmental stresses that affect their growth. Therefore, in our research, we examined the expression levels of all *PeSPL* genes under stress conditions to identify those that are involved in the responses to biotic and abiotic stress in moso bamboo. In a previous study, SA was reported to activate the transcription of many defense-related genes in response to pathogen infection [36]. In addition, SPL genes in Arabidopsis, such as *SPL3* and *SPL8*, help mediate the response to GA signaling during plant development [17]. In addition, water content is a serious environmental stress that affects the growth of moso bamboo. When water resources are limited, plants reallocate this precious resource by restricting transpiration, and they frequently flower early [70]. It is well-known that flowering will lead to death in moso bamboo. Thus, we used PEG to simulate drought conditions to observe its effect on the expression levels of *PeSPL* genes. In summary, we quantified the expression of the *PeSPL* genes in response to salicylic acid (SA), GA, and PEG (drought) treatments in moso bamboo using qRT-PCR.

Previous studies on SPL genes focused on plant growth and development by examining the relative expression in different tissues and during several stages of plant growth. To investigate the roles of SPL genes in *Phyllostachys edulis* organs and development, we also performed qRT-PCR analyses to examine the relative expression of the 32 *PeSPL* genes in young leaves, mature leaves, roots, shoots, and panicles.

**Fig. 6:**
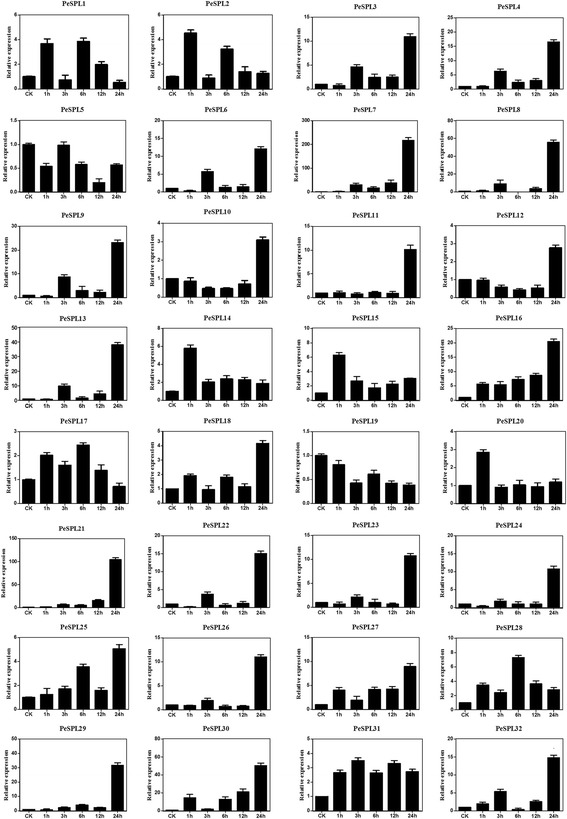
qRT-PCR expression levels of all *PeSPL* genes following SA (100 μM). The *Y-axis* indicates the relative expression levels; 0, 1, 3, 6, 9, 12, and 24 (*X-axis*) indicate hours of treatment. Mean values and standard deviations (SDs) were obtained from three biological and three technical replicates

In the SA treatment (Fig. 6), 30 *PeSPL* family genes were found to be up-regulated; however, transcription of *PeSPL5* and *PeSPL19* was reduced at all time points. We found that *PeSPL14* and *PeSPL15* were highly expressed after 1 h of treatment, and the expression of *PeSPL28* was highest at 6 h. Expression of 19 genes (*PeSPL3*, −*4*, −*6*, −*7*, −*8*, −*9*, −*11*, −*13*, −*16*, −*21*, −*22*, −*23*, −*24*, −*25*, −*26*, −*27*, −*29*, −*30*, and *32*) peaked at 24 h. *PeSPL7* showed the greatest up-regulation (by more than 230-fold), and transcription of *PeSPL21* increased >110-fold at 24 h after treatment. In addition, there were six duplicated gene pairs; *PeSPL1* and −*28*, *PeSPL4* and −*24*, *PeSPL*7 and −*13*, *PeSPL8* and −*9*, *PeSPL16* and −*29*, and *PeSPL21* and −*23*, that had similar expression profiles in response to SA treatment.

**Fig. 7:**
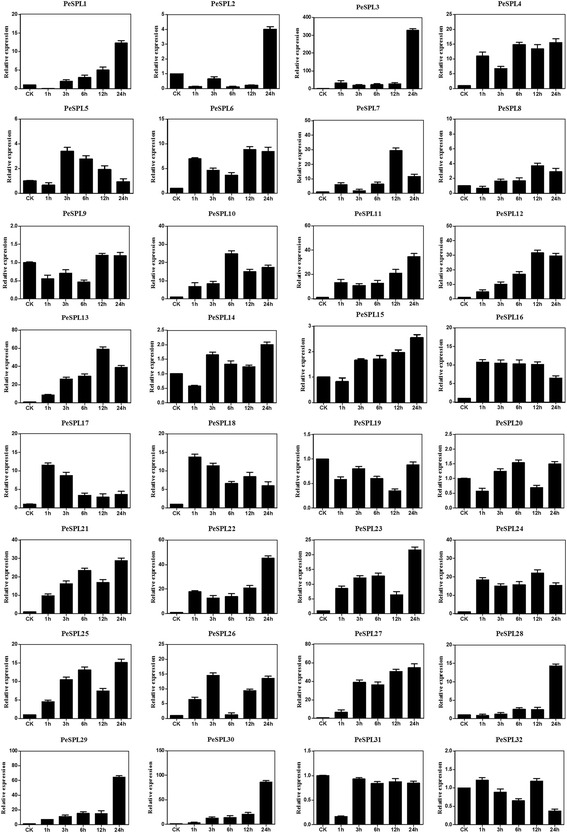
Expression analysis of *PeSPL* genes after GA induction. Sampling occurred 0, 1, 3, 6, 9, 12, and 24 h after treatment, and the relative expression levels were analyzed. Untreated sample expression levels = 1. *X-axes* represent time points after GA treatment. *Y-axes* represent relative gene expression values normalized to reference gene *TIP41*. *Bars* indicate standard deviations (SD) from three biological replicates

In the GA treatments (Fig. 7), only three paralogous pairs, *PeSPL1* and −*28*, *PeSPL7* and −*13*, and *PeSPL21* and −*23*, displayed similar expression profiles. However, differential expression patterns were observed in some duplicated gene pairs. For example, in the paralogous pair *PeSPL14* and −*30*, *PeSPL14* showed only minor changes in expression (~2-fold higher than the control), while transcription of *PeSPL30* increased nearly 100-fold over the check at 24 h. In addition, the highest expression level of *PeSPL17* occurred at 1 h, while that of *PeSPL2* occurred at 24 h. The relative expression of genes *PeSPL1*, −*3*, −*4*, −*11*, −*21*, −*22*, −*23*, −*25*, −*27*, −*28*, −*29*, and *30* peaked at 24 h after treatment, and *PeSPL3* was up-regulated by >330-fold compared to the control. Expression of three genes (*PeSPL16*, −*17* and *18*) peaked 1 h after treatment, while expression of *PeSPL6, −7, −12, −13,* and *24* also increased and the highest levels were observed at 12 h. The peak expression levels for *PeSPL10* and *PeSPL26* were observed after GA treatment for 3 and 6 h, respectively.

**Fig. 8:**
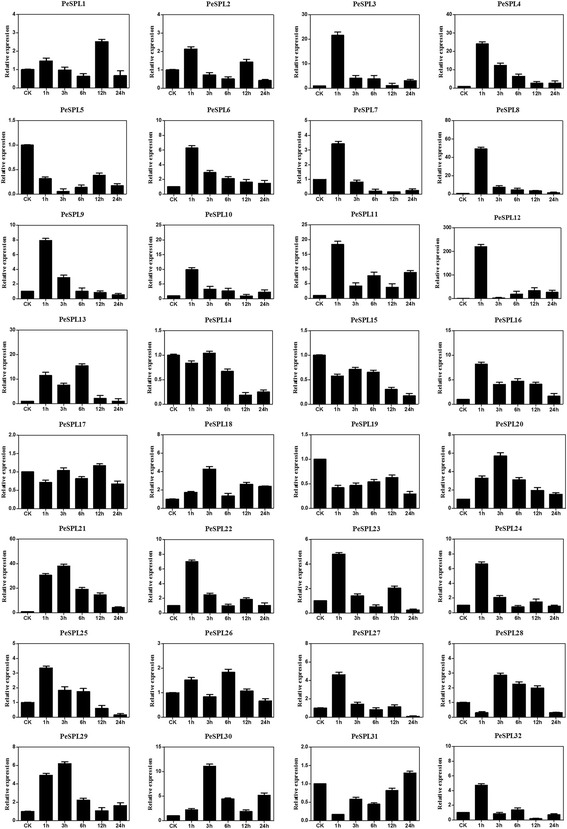
Expression patterns of all *PeSPL* genes under drought stress using qRT-PCR. Relative expression levels of 32 SPL genes were examined by qRT-PCR and normalized with respect to the reference gene *TIP41* under drought stress treatment. Bars represent standard deviations (SD) of three biological replicates. *Y-axes* indicate the scale of the relative expression levels. *X-axes* show time courses of drought stress treatments for each gene

As shown in Fig. 8 for the simulated drought treatments, transcription of all SPL genes changed in moso bamboo leaves that had been watered with the PEG solution compared to the control. Under PEG treatment, expression of *PeSPL3*, −*4*, −*6*, −*8*, −*9*, −*10*, −*11*, −*12*, −*16*, −*22*, −*23*, −*24* was induced rapidly and peaked at 1 h, whereas expression of *PeSPL29* and *PeSPL30* peaked at 3 h, and *PeSPL13* peaked at 6 h. Interestingly, expression of *PeSPL12* was strongly upregulated at 1 h by >200-fold, and then declined under drought treatment. *PeSPL5*, −*15*, and −*19* were found to be down-regulated at all five time points. Comparing the relative expression patterns of all *PeSPL* genes revealed that five paralogous gene pairs (*PeSPL4* and −*24*, *PeSPL6* and −*25*, *PeSPL8* and −*9*, *PeSPL10* and −*32*, and *PeSPL12* and −*22)* showed similar expression patterns in response to drought stress.

**Fig. 9:**
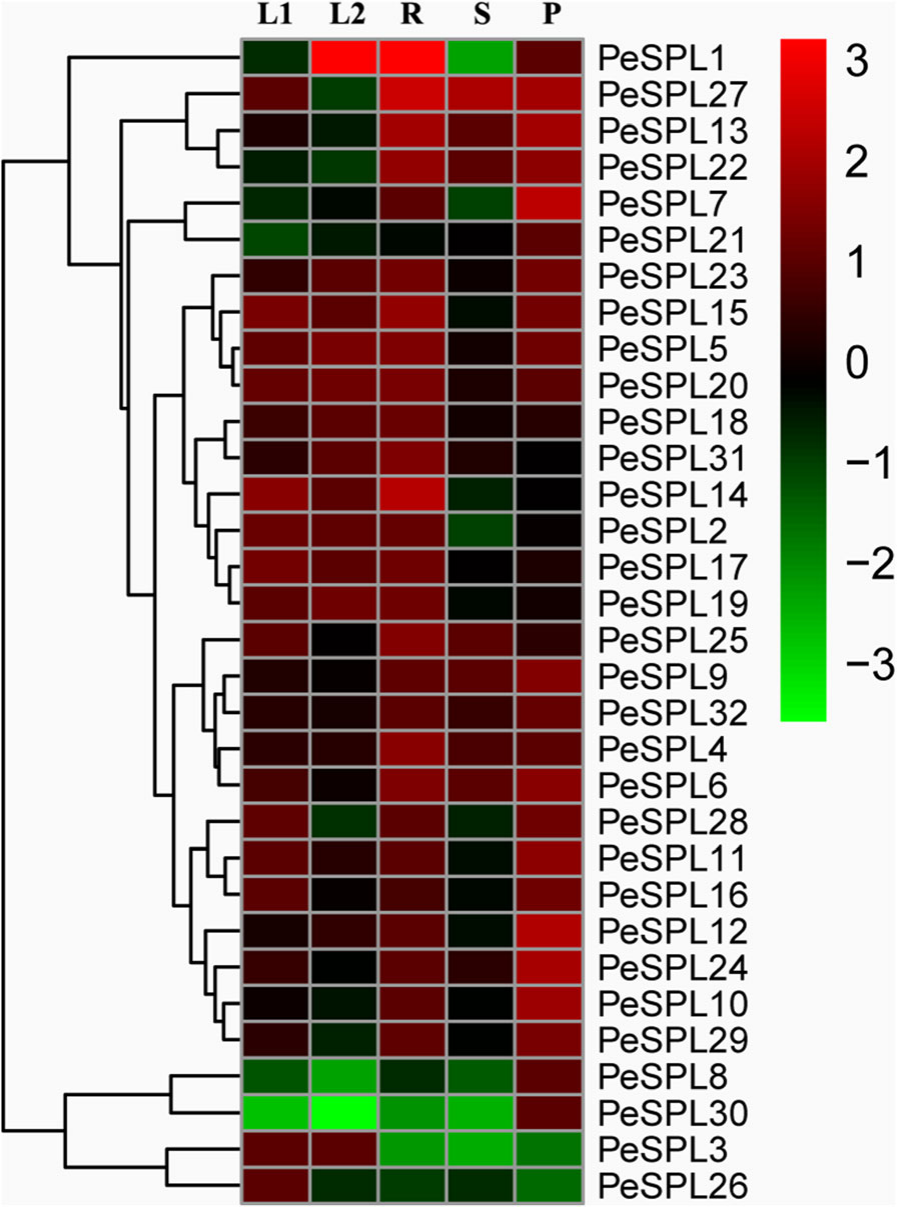
Expression analysis of *PeSPL* genes across different tissues and development stages. Sampling from the young leaf, mature leaf, root, shoot and panicle. The *green colour* indicates lower and *red* higher transcript abundance compared to the relevant control. L1: young leaf; L2: mature leaf; R: root; S: shoot; P: panicle

To predict possible functions of moso bamboo SPL genes in organ development, we determined the expression profiles of the 32 *PeSPLs* in five organs; young leaves (L1), mature leaves (L2), roots (R), shoots (S), and panicles (P) using qRT-PCR. There were a variety of transcription patterns observed for the different *PeSPLs* among the different tissues or developmental stages (Fig. 9). Twenty genes (*PeSPL4*, −*5*, −*6*, −*9*, −*10, −11*, −*12*, −*13*, −*15*, −*16*, −*17*, −*18*, −*19, −20*, −*23*, −*24*, −*25*, −*29*, −*31*, and −*32*) were expressed in all organs analyzed, while the remaining 12 genes showed dramatically different organ-specific expression in the five organs and developmental stages. Among the 32 moso bamboo SPL genes, four showed the highest mRNA accumulation in the panicle (*PeSPL7*, −*8*, −*21*, and −*30*), two in roots, shoots, and panicles (*PeSPL22* and −*27*), one in mature leaves and roots (*PeSPL1*), one in young leaves, roots, and panicles (*PeSPL28*), one in young leaves and mature leaves (*PeSPL3*), and one only in young leaves (*PeSPL26*). In addition, some paralogous gene pairs, such as *PeSPL4*/−*24*, *PeSPL5*/−*31*, *PeSPL6*/−*25*, *PeSPL10*/−*32*, and *PeSPL19*/−*20*, showed similar expression patterns. However, most of the remaining gene pairs had very different expression patterns; for instance, *PeSPL1* is highly expressed in mature leaves, while its paralog, *PeSPL28*, is expressed at a much lower level in the same tissue. Through comparisons with the heatmap, we found that most of the *PeSPL* genes had very similar expression levels in the same organs. The diverse transcription patterns observed in the different organs indicate that SPL genes in moso bamboo may play roles in the development of specific organs.

## Discussion

The SPL genes are a plant-specific transcription factor family with no homologs in bacteria, animals, or humans [20, 71]. In the present study, we performed a comprehensive analysis of the SPL gene family in *Phyllostachys edulis*, and the complex function and characteristics of SPL genes have previously been analyzed in model plant species *Arabidopsis* and rice. In our study, we identified 32 putative SPL genes in the genome of moso bamboo, which is similar to the number found in maize. Based on analysis of the phylogenetic relationships, the predicted *PeSPL* gene family was divided into eight groups (G1-G8) (Fig. 1). We found that all eight groups included different genes from rice, maize, and moso bamboo, indicating that the SPL genes had diversified prior to the evolutionary diversification of the three species. The phylogenetic tree also showed that the *PeSPL* genes grouped tightly with *OsSPL* and *ZmSBP* genes, which is consistent with the fact that maize and rice diverged from a common ancestor and are also monocots. In addition, the rice *SPL14* gene was shown to promote panicle branching and increased rice grain yield in a previous study. Therefore, it is tempting to assume that *PeSPL7*, the ortholog of *OsSPL14* in group 3 and a high expression level in panicle, is involved in the vegetative and reproductive stages in moso bamboo. For the *PeSPL* genes, analysis of their diverse intron/exon structures and protein motifs will contribute to an understanding of the different roles they play in development and growth. The *PeSPL* genes from groups 1, 4, and 7 shared similar exon-intron structures within the same phylogenetic clusters, suggesting that the evolutionary relationships of the SBP domains had a great relationship with the changing structures, which is consistent with the situation in rice [20]. The motif sequences and orders were similar for each pair of genes (Fig. 3), which showed that these gene pairs may have similar functions in moso bamboo [35]. However, we noticed that some *PeSPL* proteins had highly divergent motif patterns. For instance, motif 19 was unique to group 3 and motif 6 was only found in *PeSPL2, PeSPL15*, and *PeSPL17.* Motifs 1, 2, and 3 were highly conserved and were present in most *PeSPL* proteins, which could be important to their functions as transcription factors. Therefore, the different motifs present in the *PeSPL* proteins are most probably the structural basis for their diverse functions. Many paralogous gene pairs arise from recent gene duplication events, which are important in driving evolution and rapid expansion [72]. In addition, gene duplication events also help organisms adapt to different environments during development and growth [73, 74]. In order to explore the patterns of macroevolution and estimate the evolutionary rates in moso bamboo, we estimated Ka and Ks for the paralogous (*Pe*-*Pe*) and orthologous (*Pe*-*Os*) gene pairs and calculated Ks and the Ka/Ks ratios for each gene pair. The Ks values predicted that a large-scale duplication event occurred ~15 MYA in moso bamboo, and that the divergence times for orthologous gene pairs (*Pe*-*Os*) was approximately 34 MYA. Peng et al. showed that a whole-genome duplication event in moso bamboo occurred 7–12 MYA, and that the divergence time between *P. edulis* and rice was 7–15 MYA [37]. When compared with these results, our study indicates that the SPL gene family experienced an earlier large-scale duplication event and diversified prior to the separation of the two most recent progenitor species. In addition, Ka/Ks ratio can be used to measure the historical choice of coding sequences [75]. In general, Ka/Ks ratios >1, =1, and <1 indicate that a gene has experienced positive selection, neutral selection, and negative or purifying selection, respectively [72, 76]. Interestingly, in our study, the Ka/Ks ratios for the *Pe*-*Pe* and *Pe*-*Os* gene pairs were large at 0.45, and imply a strong selection constraint and purifying selection in the *PeSPL* genes. The analysis of SPL gene expression profiles in different tissues helps us understand the dynamics of gene expression in moso bamboo. Therefore, we used publicly available microarray data (NCBI accession number ERP001341) to analyse the gene expression profiles of the 32 SPL genes in moso bamboo. Our results suggested that most *PeSPL* genes are expressed at high levels and widely in the different organs or developmental stages that we examined. For instance, 14 of the 32 genes showed relatively high expression levels in all tissues, demonstrating that they may play important roles in processes involved in moso bamboo development and growth. In addition, we found that several *PeSPL* genes show tissue-specific expression. The mRNA levels of the 32 *PeSPL* genes in florets, leaves, and roots were significantly higher than in shoots (Fig. 5), which suggests that *PeSPLs* might be involved in the development of florets, leaves, and roots in moso bamboo.

Plant genomes contain many stress-related genes allowing plants to adapt to adverse environmental conditions. Previous studies have focused mainly on the function of the SPL gene family in development, but the expression of these genes is rarely studied under stress conditions. In our study, we found that the *PeSPL* genes are highly regulated by environmental signals and play positive roles in biotic and abiotic stress responses. The *PeSPL* genes showed significant differential expression patterns under the three biotic and abiotic stress conditions tested. Most *PeSPL* genes were upregulated by the three stress treatments, suggesting that *PeSPL* genes may play crucial roles in regulating stress responses in moso bamboo. For example, the high expression of *PeSPL7*, −*8*, −*13*, −*21*, −*29*, −*30* indicated that they may play essential role in response to pathogen infection (SA). In addition, *PeSPL3*, −*7, −10, −11, −12, −13*, −*22*, −*27*, −*29*, −*30* and *PeSPL3, −4, −8, −12, −21* may also have function in response to GA signaling and drought (PEG), respectively. We also found that some genes did not respond to these stresses, especially *PeSPL19*, which had low expression levels in response to the SA, GA, and PEG treatments. Furthermore, most of the paralogous gene pairs had similar expression levels and patterns under the three stress treatments in the same paralogous pair. These results may indicate that homologous genes have similar functions in the processes of organismal growth and development. Expression profiles of the 32 *PeSPL*s in five tissues showed that most of these genes were expressed at high levels in all tissues. Previous studies demonstrated that SPLs play vital roles in flower development [71, 77], and *miR 156* target the SPL transcription factors that cooperate to regulate bamboo flowering [78]. In our research, we found that most SPL genes in moso bamboo showed higher expression levels in the panicle. Except for *PeSPL3* and *PeSPL26*, all of the *PeSPL* genes exhibited relatively high transcript levels in the flower (Fig. 9), implying that these genes are important in flower development. In addition, *miR 156* target the SPL transcription factors that cooperate to regulate bamboo flowering. Unlike most other plants, flowering is an unpredictable and uncontrollable event in moso bamboo, and the plants die after they flower [2]. The results of our study suggest that expression of *PeSPLs* in flowers provides a basic understanding for further investigations into the flowering mechanism in moso bamboo.

## Conclusions

The results here represent the first genome-wide analysis of the SPL gene family in moso bamboo. We systematically analyzed the 32 predicted *PeSPL* genes, including their gene structure, phylogeny, conserved motifs, promoter regions, gene duplication, and expression profiling, which may be related to their biological functions. The phylogenetic analysis clustered these SPL genes into eight groups. In each group, the motif compositions and exon/intron structure were fairly well conserved. Furthermore, the expression patterns of *PeSPL* genes show that they play potentially important roles in mediating the effects of stress induced by drought (PEG), SA (pathogen infection), and GA. In addition, the expression patterns in various tissues show that *PeSPL* genes may function in moso bamboo growth and development. The results of our study establish a foundation for future studies on the functions of SPL genes in organ development and the plant stress response, and provide a basic understanding that may allow us to further elucidate the potential functions of the *PeSPL* genes in moso bamboo.

## Additional file

Additional file 1: Table S1. Oligonucleotide primers used in qRT-PCR assays for all 32 *PeSPL* genes. **Table S2.** Gene names and locus information for the SPL proteins in rice and maize. **Table S3.** Protein sequences and lengths of the major motifs identified by MEME in the putative *PeSPL* proteins. **Table S4.** Nucleotide substitution rates for the paralogous SPL gene pairs identified in the moso bamboo genome. **Table S5.** Nucleotide substitution rates for the orthologous SPL gene pairs between moso bamboo and rice. **Table S6.** Summary of abiotic stress-inducible cis-elements in the promoter regions of SPL genes in moso bamboo. **Table S7.** Microarray expression data for the 32 SPL genes in moso bamboo. These primary data was downloaded from NCBI, and then the relative expression level (log10 expression values) of 7 different issues or development stages was obtained after a series of manual processing. L, leaf; P1, early panicle; P2, advanced panicle; R, root; Rh, rhizome; S1, 20-cm shoot; S2, 50-cm shoot. (ZIP 104 kb)

## Abbreviations

AP1: APETALA1
CDS: Coding sequence
GA: Gibberellin
GDB: Genome database
GSDS: Gene structures display server
Ka: The rate of nonsynonymous substitutions
Ks: The rate of synonymous substitutions
MYA: Million years ago
NCBI: National Center of Biotechnology Information
NCGR: National Center for Gene Research
N-J: Neighbour-Joining
PEG: Polyethylene glycerol
qRT-PCR: Quantitative real-time reverse transcription PCR
RNA-seq: RNA sequencing
SA: Salicylic acid
SBP: *SQUAMOSA* promoter binding protein
SMART: Simple Modular Architecture Research Tool
SPL: *SQUAMOSA* promoter binding protein-like
SRA: Short read archive
TFs: Transcription factors
TIP: Tonoplast intrinsic protein
