# Supplementary material for: Genome-wide identification and expression analysis of SBP-like transcription factor genes in Moso Bamboo (Phyllostachys edulis)
Source: BMC Genomics. 2017 Jun 27;18:486. doi: 10.1186/s12864-017-3882-4 (PMC5488377; doi:10.1186/s12864-017-3882-4)
Supplement: Additional file 1: Table S1. — Oligonucleotide primers used in qRT-PCR assays for all 32 PeSPL genes. Table S2. Gene names and locus information for the SPL proteins in rice and maize. Table S3. Protein sequences and lengths of the major motifs identified by MEME in the putative PeSPL proteins. Table S4. Nucleotide substitution rates for the paralogous SPL gene pairs identified in the moso bamboo genome. Table S5. Nucleotide substitution rates for the orthologous SPL gene pairs between moso bamboo and rice. Table S6. Summary of abiotic stress-inducible cis-elements in the promoter regions of SPL genes in moso bamboo. Table S7. Microarray expression data for the 32 SPL genes in moso bamboo. These primary data was downloaded from NCBI, and then the relative expression level (log10 expression values) of 7 different issues or development stages was obtained after a series of manual processing. L, leaf; P1, early panicle; P2, advanced panicle; R, root; Rh, rhizome; S1, 20-cm shoot; S2, 50-cm shoot. (ZIP 104 kb) [file 12864_2017_3882_MOESM1_ESM.zip › additional file 1/Table S4.docx]

| **Table S4**. Nucleotide substitution rates for the paralogous SPL gene pairs identified in the moso bamboo genome. | | | |
| --- | --- | --- | --- |
| **paralogous pairs** | **Ka value** | **Ks value** | **Ka/Ks** |
| *PeSPL1-PeSPL28* | 0.11696 | 0.2076 | 0.563391137 |
| *PeSPL2-PeSPL17* | 0.04259 | 0.15104 | 0.281978284 |
| *PeSPL3-PeSPL13* | 0.14023 | 0.26154 | 0.536170375 |
| *PeSPL4-PeSPL24* | 0.02278 | 0.09828 | 0.231786732 |
| *PeSPL5-PeSPL31* | 0.03286 | 0.12826 | 0.256198347 |
| *PeSPL6-PeSPL25* | 0.02772 | 0.12513 | 0.221529609 |
| *PeSPL6-PeSPL29* | 0.23135 | 0.6131 | 0.377344642 |
| *PeSPL7-PeSPL13* | 0.17719 | 0.21378 | 0.828842736 |
| *PeSPL8-PeSPL9* | 0.04238 | 0.12396 | 0.341884479 |
| *PeSPL10-PeSPL32* | 0.10065 | 0.10492 | 0.959302326 |
| *PeSPL12-PeSPL22* | 0.19754 | 0.20874 | 0.946344735 |
| *PeSPL14-PeSPL30* | 0.02166 | 0.09022 | 0.240079805 |
| *PeSPL16-PeSPL29* | 0.11027 | 0.16371 | 0.673569116 |
| *PeSPL19-PeSPL20* | 0.09345 | 0.2049 | 0.456076135 |
| *PeSPL21-PeSPL23* | 0.10667 | 0.20333 | 0.524615158 |
| *PeSPL25-PeSPL29* | 0.19656 | 0.59679 | 0.329362087 |

The statistical data of the *Pe-Pe*

| **Ks value** | numbers | frequency | **Ka/Ks** | numbers | frequency |
| --- | --- | --- | --- | --- | --- |
| 0-0.1 | 2 | 0.125 | 0-0.1 | 0 | 0 |
| 0.1-0.2 | 6 | 0.375 | 0.1-0.2 | 0 | 0 |
| 0.2-0.3 | 6 | 0.375 | 0.2-0.3 | 5 | 0.3125 |
| 0.3-0.4 | 0 | 0 | 0.3-0.4 | 3 | 0.1875 |
| 0.4-0.5 | 0 | 0 | 0.4-0.5 | 1 | 0.0625 |
| 0.5-0.6 | 1 | 0.0625 | 0.5-0.6 | 3 | 0.1875 |
| 0.6-0.7 | 1 | 0.0625 | 0.6-0.7 | 1 | 0.0625 |
|  |  |  | 0.7-0.8 | 0 | 0 |
|  |  |  | 0.8-0.9 | 1 | 0.0625 |
|  |  |  | 0.9-1.0 | 2 | 0.125 |
